# Exercise-induced reductions in central command network cerebral blood flow assessed with arterial spin labeling MRI

**DOI:** 10.1162/imag_a_00386

**Published:** 2024-12-19

**Authors:** Jessica Cloud, Jessica Stark, Alexander N. Hasselbach, Kelly J. Hiersche, David H. Salat, Meher R. Juttukonda, Scott M. Hayes

**Affiliations:** Department of Psychology, The Ohio State University, Columbus, OH, United States; Athinoula A. Martinos Center for Biomedical Imaging, Department of Radiology, Massachusetts General Hospital, Charlestown, MA, United States; Neuroimaging Research for Veterans Center, VA Boston Healthcare System, Boston, MA, United States; Department of Radiology, Harvard Medical School, Boston, MA, United States; Chronic Brain Injury Initiative, The Ohio State University, Columbus, OH, United States

**Keywords:** arterial transit time, exercise, fitness, physical activity, perfusion, cerebral blood flow

## Abstract

Habitual aerobic exercise positively impacts brain health. However, less is known about the acute effects of exercise on regional cerebral blood flow (CBF). Most studies have used exercise methods that required a 10-minute or longer delay before acquiring CBF data or methods that assessed global, rather than regional, CBF. In the current feasibility study, an aerobic exercise stimulus was administered using an MRI-compatible cardio step module connected to the MRI table. This allowed participants (n = 12, mean age = 20 years) to exercise at light-to-moderate intensity while in the bore of the MRI scanner. Pseudo-continuous arterial spin labeling MRI with multiple delays was collected on a 3T Siemens MRI scanner immediately pre- and post-exercise. CBF and arterial transit time changes were examined within the central command network, which impacts motor and cardiovascular systems during exercise. Associations with perceived exertion and cardiorespiratory fitness were assessed. CBF in central command regions decreased following exercise, with greater decreases in regions associated with cardiovascular control. For example, CBF in the left insula decreased by -9.03 ± 8.69 mL/100 g/min (-11.45%,*p*< 0.01); CBF in the left and right rostral anterior cingulate decreased by -4.91 ± 6.08 mL/100 g/min (-6.17%,*p*= 0.02) and by -5.04 ± 6.34 mL/100 g/min (-7.43%,*p*= 0.02); and CBF in the left lateral orbitofrontal cortex decreased by -7.30 ± 8.11 mL/100 g/min (-9.50%,*p*= 0.01). Decreased CBF was also associated with greater ratings of perceived exertion in cardiovascular command regions, including right insula (r = -0.67,*p*= 0.03), medial orbitofrontal cortex (r = -0.64,*p*= 0.04), and lateral orbitofrontal cortex (r = -0.75,*p*= 0.01). The current study further demonstrates the feasibility of assessing CBF immediately following exercise using an exercise stimulus in the bore of the MRI scanner. These results contribute to a small but growing body of literature describing cerebral hemodynamics immediately following exercise.

## 1 Introduction

Neural and cognitive function are impacted by variables related to the function of the cardiorespiratory system (heart, vascular, and lung) (Alosco & Hayes, 2015;Hayes et al., 2016;Maleki et al., 2022;Salzman et al., 2022). In the brain, these relationships can be examined at rest with longitudinal design—for instance, by completing a baseline brain magnetic resonance imaging (MRI) scan while the participant is at rest, implementing a 6-month exercise intervention, and then repeating MRI scanning at rest following intervention. However, cardiac, vascular, and respiratory functions change dynamically in response to acute physiological demands (e.g., aerobic exercise), which is not captured by the brain-at-rest imaging approach. By scanning during or shortly after the cardiorespiratory system is challenged by an exercise stimulus, however, exercise-induced alterations in the brain can be assessed.

A variety of neuroimaging methods have been used to examine the acute effects of exercise on cerebral perfusion. Initial studies using Xenon clearance concluded that exercise does not influence cerebral hemodynamics, for example,Globus et al. (1983). However, this method takes over 10 minutes to acquire a single image of cerebral blood flow (CBF) and therefore is relatively insensitive to transient alterations in cerebral hemodynamics. Other methods have significantly better temporal resolution, but at the cost of spatial resolution. For instance, alterations in CBF during compound muscle exercise have been assessed with transcranial doppler ultrasonography (TCD) (Braz & Fisher, 2016;Hellstrom et al., 1996;Imray et al., 2005;Linkis et al., 1995) or functional near-infrared spectroscopy (fNIRS) (González-Alonso et al., 2004;Rooks et al., 2010). Studies with both modalities have reported increased CBF during exercise (Braz & Fisher, 2016;Hellstrom et al., 1996;Mulser & Moreau, 2023;Rooks et al., 2010); once exercise reached an intensity threshold, CBF decreased, sometimes back to baseline levels (Braz & Fisher, 2016;González-Alonso et al., 2004;Hellstrom et al., 1996;Mulser & Moreau, 2023;Rooks et al., 2010). However, these studies were not able to assess regional variability throughout the cortical mantle or in subcortical regions due to limitations in the spatial resolution of TCD and fNIRS.

In contrast, arterial spin labeling (ASL)-MRI is capable of higher spatial resolution imaging, which allows for regional assessment of CBF and other flow-related metrics of hemodynamics. However, MRI is not often used to assess acute exercise effects as participants lay supine on a table in the bore of the MRI scanner during data acquisition, limiting the type of exercise stimulus that can be administered. A common solution to this problem is to implement the exercise stimulus (treadmill or cycle-based exercise protocol) outside the bore of the magnet, and then the participant is moved into the bore of the scanner for subsequent brain imaging. Studies using this approach in healthy adults typically observed decreased CBF in gray matter regions compared to resting state (MacIntosh et al., 2014;Smith et al., 2010; but seeSteventon et al., 2020). Administering the exercise stimulus outside the bore of the scanner, however, introduces a delay between the exercise stimulus and CBF assessment: transition from the exercise equipment to the MRI table, getting the participant comfortable, landmarking the participant, collecting the localizer scan, running the ASL calibration scan, and then starting ASL-MRI scanning typically takes about 10 minutes. Therefore, most studies utilizing higher-resolution imaging methods do not acquire CBF data until 10–60 minutes after the exercise stimulus (MacIntosh et al., 2014;Smith et al., 2010;Steventon et al., 2020). This delay limits insight into dynamic alterations in brain function immediately subsequent to an exercise stimulus.

In the current feasibility study, we used an MRI-compatible cardio step module to administer a lower body compound-muscle exercise stimulus in the bore of the MRI scanner, substantially reducing the delay between the exercise stimulus and ASL-MRI data acquisition to about 90 seconds and capturing cerebral blood flow within 10 minutes of exercise cessation (end of exercise stimulus to end of ASL scan). To our knowledge, one study to date has administered a similar “in-scanner” exercise protocol and assessed CBF with ASL-MRI (Mast et al., 2022). Using a pseudo-continuous ASL sequence with a single post-labeling delay (TR = 4550 ms; spatial resolution = 2.75 x 2.75 x 5 mm), they found that whole-brain and regional CBF decreased following exercise. The current study extends the work ofMast et al. (2022)by utilizing pseudo-continuous ASL-MRI with multiple post-labeling delays, which renders CBF less sensitive to changes in arterial transit time (ATT), and higher spatial resolution, which mitigates the potential for partial volume effects (Fan et al., 2019;Juttukonda et al., 2021). It also allows for computation of additional measures of cerebral hemodynamics, such as ATT, which captures the time taken for labeled blood to reach brain tissue and which, to our knowledge, has not been previously reported in the context of exercise physiology. We leverage this state-of-the-art sequence to examine CBF and ATT in the central command network, brain regions that govern motor and cardiovascular systems during performance of exercise. Finally, we consider the changes in cerebral hemodynamics within the context of relevant performance variables—cardiorespiratory fitness and perceived exertion—to probe what factors may underlie changes in cerebral hemodynamics with exercise.

## 2 Methods

### 2.1 Participants

Young adults (n = 12; 6 males; age 18–22 years) were recruited from The Ohio State University community. Exclusion criteria included: history of serious illness including seizures, cerebrovascular accident, myocardial infarction, and type 1 diabetes; current active suicidal or homicidal ideation, intent, or plan requiring crisis intervention; current DSM diagnosis of bipolar disorder, schizophrenia, or any other psychotic or cognitive disorder; and contraindications to MRI or exercise without the supervision of a physician (based on American College of Sports Medicine (ACSM) guidelines and assessed with the Physical Activity Readiness Questionnaire, PAR-Q;Canadian Society for Exercise Physiology, 2002;Thomas et al., 1992). No exclusions were made based on ethnic or racial background. All experimental procedures were approved by The Ohio State University Institutional Review Board. Written informed consent was obtained from all participants, and all participants received financial compensation for participation.

Prior to testing, participants reported general health status on a scale of “poor” to “excellent” and reported weekly physical activity using the International Physical Activity Questionnaire (IPAQ), Long Form (Craig et al., 2003).

### 2.2 Cardiopulmonary exercise testing

Progressive graded maximal exercise testing was conducted using an Excalibur Sport cycle ergometer with 2-minute stages of increasing resistance and a pedal speed of 60 revolutions per minute. Peak rate of oxygen consumption (VO_2_peak, mL/kg/min) and respiratory exchange ratio (RER) were measured across 30-second time windows. Heart rate, blood pressure, and ratings of perceived exertion (RPE) using the 20-item Borg scale (Borg, 1998), which assesses physical exertion on a scale of 6 (no exertion at all) to 20 (maximal exertion), were assessed every 2 minutes, in time with each phase change. Participants were asked to continue cycling to failure. VO_2_peak was considered valid if at least two of the following criteria were met: (a) RER > 1.1; (b) achieved maximum heart rate equivalent to 85% of their age-predicted maximum (HR_max_; 220 - age); and (c) RPE > 17, which corresponds to an exertion level of “very hard.”

### 2.3 Magnetic resonance imaging

#### 2.3.1 Image acquisition

Images were acquired on a Siemens Prisma 3T scanner (Siemens Healthineers; Erlangen, Germany) with a 32-channel head coil using acquisition parameters consistent with the Lifespan Human Connectome Project (Harms et al., 2018). T_1_-weighted multi-echo magnetization-prepared rapid gradient echo (ME-MPRAGE) (repetition time (TR) = 2500 ms; echo time (TE) = 1.8, 3.6, 5.4, 7.2 ms; inversion time (TI) = 1010 ms; spatial resolution = 0.8 x 0.8 x 0.8 mm; multiband factor = 4) was collected, followed by pseudo-continuous ASL (TR = 3600 ms; TE = 20 ms; spatial resolution = 2.5 mm^3^; labeling duration = 1500 ms; post-labeling delays [control/label pairs] = 200 ms [6 pairs], 700 ms [6 pairs], 1200 ms [6 pairs], 1700 ms [10 pairs], 2200 ms [15 pairs]). The exercise stimulus was then administered during collection of blood oxygenation level dependent (BOLD) functional MRI (results not reported here). Following exercise, post-exercise pseudo-continuous ASL was repeated. Time from the end of the exercise stimulus to the end of the post-exercise ASL scan was no greater than 10 minutes. Finally, T_2_-weighted images (TR = 3200 ms; TE = 564 ms; spatial resolution = 0.8 x 0.8 x 0.8 mm) were collected.

#### 2.3.2 Exercise stimulus

**Fig. 1.**
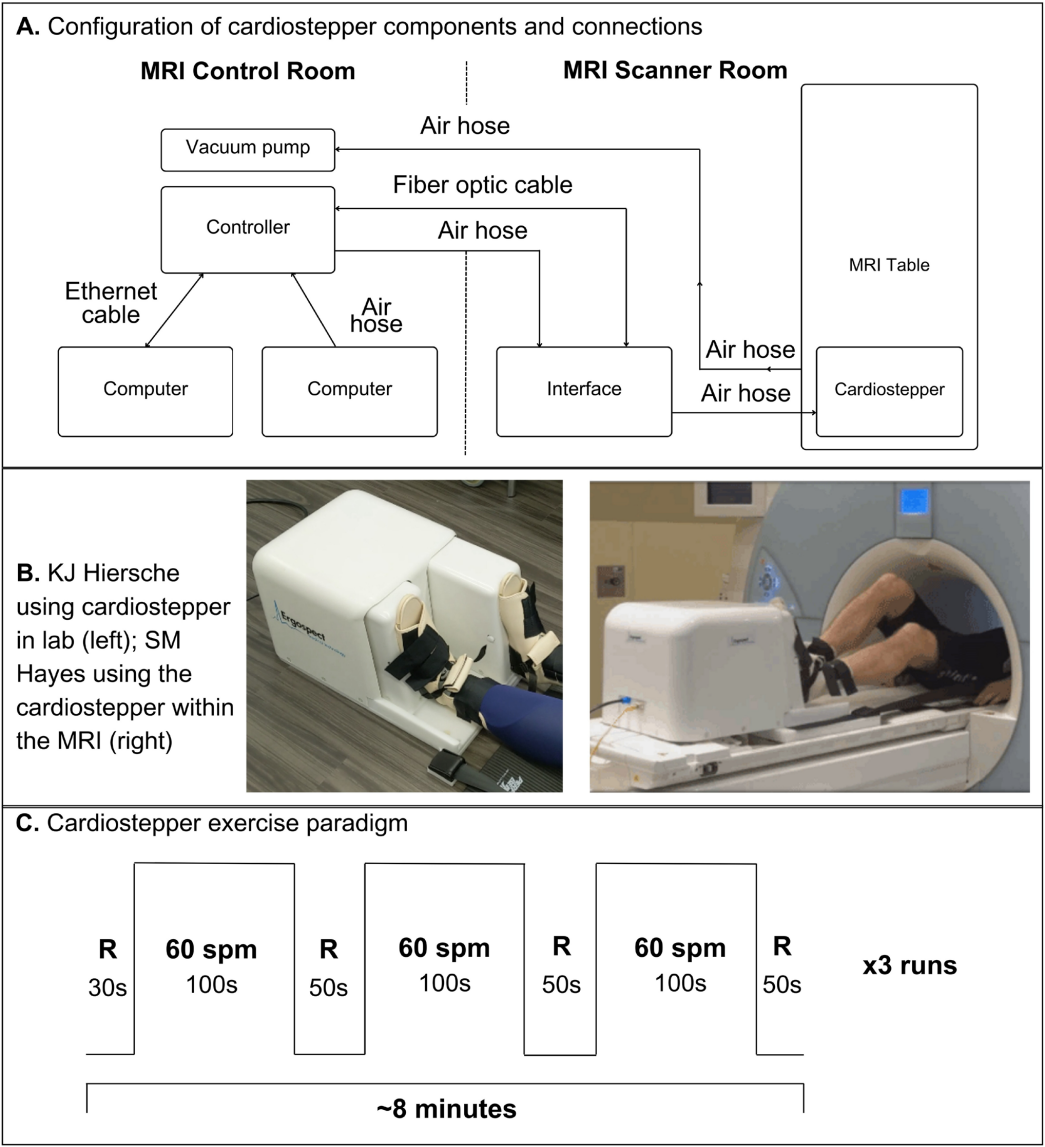
(A) Configuration of cardio step module components and connections. The cardio step module is secured to the MRI table and connected via air hose to the vacuum pump and controller (via the cardio step module interface). The controller-interface system is connected via air hose to an air compressor and via ethernet to the computer (which governs stepping intensity and receives information about stepping speed). (B) KJ Hiersche using the cardio step module in the lab (left); SM Hayes using the cardio step module within the MRI (right). When using the cardio step module, participants have two points of connection, limiting movement and ensuring that contact between the participant and cardio step module is not lost. Velcro straps secure the participant’s feet to the pedals. A shoulder harness is used to prevent participant’s body from pushing away from the device while stepping. (C) Participants performed three sequential runs of exercise. During each run, participants alternated between rest (R) and stepping at 60 steps per minute (spm), resulting in 15 total minutes of exercise.

The exercise stimulus (Fig. 1) was administered using the cardio step module from ErgoSpect Medical Technology (Innsbruck, Austria). This is an MRI-compatible Stairmaster-like device which can be used to generate an aerobic exercise stimulus. The module was attached to the MRI table and stabilized using suction from an oil-less Becker model VT 4.4 rotary vane vacuum pump and motor, located in the control room. Pedal resistance was generated by an air compressor (Kobalt Quiet Tech 26-gallon portable electric vertical air compressor), with the amount of resistance controlled by ErgoSpect computer software (Fig. 1A).

Participants were instructed to lay supine on the MRI table and were fitted with ear plug-style headphones, a pulse oximeter, and a respiration belt. Additionally, participants were fitted with an adjustable vest which was connected to the cardio step module with straps. The vest and straps provided some stability to the participant during exercise, as it ensured the participant did not increase their vertical distance from the unit while stepping. Participants’ feet were secured to the cardio step module pedals with adjustable Velcro straps (Fig. 1B). Their heads were stabilized with cushions to reduce head motion. Next, participants completed a practice exercise block prior to MRI scanning. The practice was designed to (a) identify a challenging resistance range for each participant and (b) allow participants to practice stepping at the desired rate. To assist participants with the rate of stepping, an audio stimulus of a metronome at 100 beats per minute was presented to participants throughout practice and task administration.

The exercise stimulus was administered using the cardio step module during a series of functional MRI scans (results not reported here). Participants completed three functional runs. Each run was composed of alternating rest and stepping blocks for a total of 8:15 minutes. Participants were given audio cues to indicate when to step and when to rest. Each block of stepping began at low resistance, followed by 30 seconds during which resistance increased every 5 seconds until the desired maximal resistance was reached. Maximal resistance stepping was maintained for 30 seconds followed by 30 seconds of low resistance stepping and 30 seconds of rest before the next block began. Each run included three stepping blocks (Fig. 1C). Heart rate and head motion was monitored in real time and, following each run, participants rated their perceived exertion using the 20-item Borg scale. Stepping resistance was assessed between each run and increased if the participant did not reach 60% of their HR_max_; or decreased if (a) the participant spent more than half of the previous run with a heart rate greater than 80% of their HR_max_(moderate intensity exercise), (b) the participant at any point in the previous run approached or exceeded their HR_max_, or (c) significant head motion was observed (framewise displacement > 0.6 mm).

#### 2.3.3 Image processing and analyses

All image processing was performed via the Ohio Supercomputer Center, which provides high-performance computing services to academic researchers from across the state of Ohio (Ohio Supercomputer Center, 1987). Cortical reconstruction and volumetric segmentation were performed with the FreeSurfer image analysis suite (version 7.2.0,http://surfer.nmr.mgh.harvard.edu/) using the T_1_- and T_2_-weighted images. Raw T_1_-weighted MPRAGE images and structural segmentation quality were assessed both visually and via Euler number (Rosen et al., 2018).

Motion correction and computation of cerebral blood flow (CBF; mL/100 g/min) and arterial transit time (ATT; seconds) were performed on ASL data using the Bayesian Inference for Arterial Spin Labeling (BASIL) toolbox (version 6.0.4; FMRIB Software Library, Oxford, UK), which produces voxel-wise maps of CBF and ATT (Chappell et al., 2009). CBF and ATT maps were registered to FreeSurfer anatomical space using FreeSurfer’s*bbregister*.

Region of interest (ROI) analysis was performed by extracting average ROI values using the Desikan-Killiany-Tourville parcellation (Desikan et al., 2006). Eight ROIs associated with the central command network (medial and lateral orbitofrontal cortex, caudal and rostral anterior cingulate cortex, posterior cingulate cortex, insular cortex, and precentral and postcentral gyrus) and two control regions not associated with the central command network (frontal pole, whole-brain gray matter) were analyzed, with each hemisphere assessed separately (18 total regions). Changes in CBF and ATT were computed by subtracting the pre-exercise from the post-exercise ROI average value in each region, such that positive values indicate greater perfusion or longer arrival time following exercise. Paired-samples t-tests were performed for each ROI.

For surface-based analyses, maps were first further registered to FreeSurfer’s*fsaverage*standard surface space. Freesurfer’s*mri_glmfit*was used to assess the group mean difference in CBF and ATT, with analyses limited to regions associated with the central command network. In this step, correction for multiple comparisons using Monte Carlo simulations was performed to identify significant clusters, using a cluster-forming threshold and significance threshold of*p*= 0.05.

Associations of VO_2_peak and RPE with differences in pre-exercise and post-exercise values were assessed using Pearson’s correlations in each of the eight pre-defined central command network ROIs (medial and lateral orbitofrontal cortex, caudal and rostral anterior cingulate, posterior cingulate cortex, insular cortex, and precentral and postcentral gyrus). Given the smaller sample size due to focus on establishing feasibility, correction for multiple comparisons was not performed in this step.

#### 2.3.4 Physiological data collection and processing

Siemens 3T Prisma pulse oximeter and respiratory belt were used to collect physiological data during the scan session. Raw pulse oximeter signals during stepping runs and ASL scan collection were processed in MATLAB (version 2019b). Signal peaks corresponding to each pulse were identified, and all pulse-to-pulse intervals were calculated and converted to beat-to-beat heart rate values. The beat-to-beat time series were then median smoothed (kernel = 5), and instantaneous beats per minute (BPM) were estimated at each TR by averaging the smoothed beat-to-beat values with a 5-second window before the TR. Peak BPM was identified for each run, and peak percent of HR_max_was calculated.

## 3 Results

### 3.1 Participants

All participants self-reported “good” (n = 7) or “excellent” (n = 5) general health and reported participating in physical activity for 120–525 minutes per week (mean minutes = 305, SD = 102). Cardiorespiratory fitness ranged from “poor” to “excellent” (mean VO_2_peak = 37.83, SD = 11.45) (American College of Sports Medicine et al., 2010). No participants were excluded from analyses due to poor image quality (mean Euler = -18.17, SD = 9.48).

### 3.2 Protocol validation

During the exercise protocol, participants reached an average maximum heart rate of 117.7 BPM (SD = 29.0, range = 75.5–168.14), which corresponds to 60% (SD = 10%, range = 37%–84%) of their HR_max_, and an average RPE of 12.8 (SD = 2.6, range = 9–16.8), corresponding to “somewhat hard” effort. These objective and subjective measures of exercise intensity indicate that the participants were exercising at a light-to-moderate intensity on average (American College of Sports Medicine et al., 2010).

Following cessation of exercise, pseudo-continuous ASL image acquisition began on average 2 minutes 32 seconds later (SD = 30 seconds). This delay included 50 seconds of rest at the end of the exercise block during which fMRI data were acquired. The maximum delay from exercise cessation to the start of ASL image acquisition for any participant was 3 minutes 18 seconds.

### 3.3 Exercise-induced changes in cerebral perfusion

Example CBF images and mean difference post-labeling delay curves are shown inFigure 2. Before exercise, average whole-brain gray matter CBF across all participants was 72.33 mL/100 g/min, consistent with CBF values in grey matter from studies with similar ASL sequences (Juttukonda, et al., 2021;Woods, et al., 2020;Yetim, et al., 2023). ROI analyses found decreases in 5 of the 18 examined gray matter regions of the central command network following exercise: left insular cortex, left lateral orbitofrontal cortex, left and right rostral anterior cingulate, and right caudal anterior cingulate (Table 1;Fig. 3A). Two additional regions showed trending changes in CBF following exercise: the left precentral gyrus (*p*= 0.089) and left postcentral gyrus (*p*= 0.051). No other examined regions showed significant change in CBF post-exercise.

**Fig. 2.**
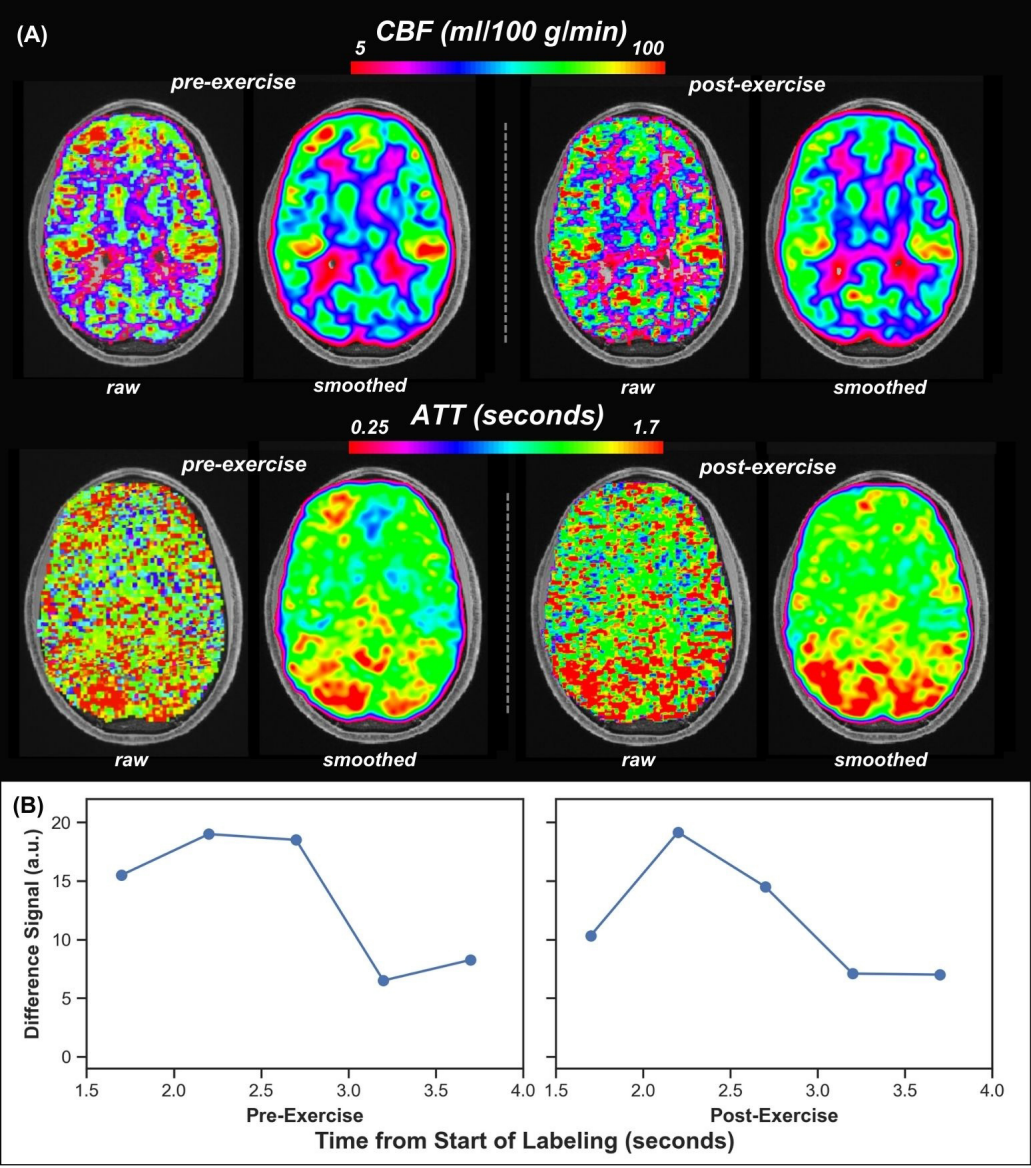
(A) Example CBF and ATT maps pre-exercise and post-exercise from a single, representative subject. In addition to CBF and ATT images without any smoothing (raw), which are the basis for the quantifications reported in this manuscript, images smoothed with a 5 mm kernel are also shown for visualization of the expected contrasts in CBF and ATT images*.*(B) Mean control label difference signal time courses for a representative voxel in the left insula pre-exercise and post-exercise.

**Table 1.** Paired-samples T-tests on the change in CBF (mL/100 g/min) and ATT (seconds) before and after light-to-moderate intensity exercise.

Region			Pre-exercise	Post-exercise					
Frontal	Measure	Hemi	*M*	*SD*	*M*	*SD*	Δ	*t* (11)	*p*
Anterior cingulate (caudal)	CBF	L	81.70	12.51	78.88	11.71	-2.83	-1.27	0.229
		**R**	**76.59**	**15.50**	**71.28**	**10.55**	**-5.32**	**-2.64**	0.047 **
	ATT	L	1.07	0.13	1.08	0.10	-0.01	0.35	0.733
		R	1.17	0.18	1.17	0.15	-0.00	-0.13	0.899
Anterior cingulate (rostral)	CBF	**L**	**75.22**	**15.79**	**70.31**	**15.06**	**-4.91**	**-2.67**	0.022 **
		**R**	**63.89**	**13.31**	**58.85**	**12.51**	**-5.04**	**-2.64**	0.023 **
	ATT	L	1.10	0.14	1.11	0.10	0.01	-0.21	0.838
		R	1.17	0.13	1.16	0.10	-0.01	-0.38	0.713
Frontal pole	CBF	L	65.26	16.83	65.90	15.61	0.64	0.19	0.848
		R	61.78	17.90	61.94	12.12	0.15	0.05	0.959
	ATT	L	1.25	0.15	1.25	0.13	-0.00	-0.18	0.859
		R	1.16	0.14	1.20	0.15	0.04	0.74	0.472
Orbitofrontal cortex (lateral)	CBF	**L**	**80.97**	**12.88**	**73.67**	**16.67**	**-7.30**	**-2.98**	-.012 **
		R	77.04	19.64	75.36	14.45	-1.67	-0.33	0.749
	ATT	L	1.21	0.09	1.21	0.08	-0.01	-0.24	0.812
		R	1.15	0.12	1.15	0.11	0.00	0.01	0.992
Orbitofrontal cortex (medial)	CBF	L	77.07	15.12	74.73	14.43	-2.34	-0.77	0.459
		R	57.26	14.02	55.56	11.06	-1.69	-0.57	0.582
	ATT	L	1.20	0.09	1.19	0.07	-0.01	-0.48	0.642
		R	1.24	0.11	1.24	0.08	0.00	0.09	0.929

Parietal			*M*	*SD*	*M*	*SD*			
Insular cortex	CBF	**L**	**80.56**	**13.96**	**71.52**	**16.21**	**-9.04**	**-3.45**	0.005 **
		R	69.10	17.17	70.74	13.44	1.64	0.37	0.719
	ATT	L	1.11	0.14	1.11	0.10	0.00	0.11	0.914
		R	1.10	0.13	1.13	0.11	0.03	0.88	0.394
Precentral gyrus	CBF	**L**	**80.94**	**15.93**	**76.33**	**18.66**	**-4.61**	**-1.86**	0.089 *
		R	76.62	20.09	77.25	19.07	0.63	0.23	0.821
	ATT	L	1.29	0.14	1.34	0.14	0.04	1.07	0.306
		**R**	**1.22**	**0.12**	**1.28**	**0.09**	**0.06**	**2.51**	0.029 **
Postcentral gyrus	CBF	**L**	**73.50**	**12.28**	**68.15**	**16.40**	**-5.35**	**-2.19**	0.051 *
		R	68.69	19.02	71.31	17.35	2.62	0.79	0.448
	ATT	L	1.32	0.11	1.34	0.11	0.02	0.51	0.623
		**R**	**1.23**	**0.11**	**1.29**	**0.07**	**0.06**	**2.39**	0.036 **
Posterior cingulate	CBF	L	87.35	17.44	82.38	14.70	-4.97	-1.61	0.135
		R	89.08	17.09	84.49	15.44	-4.59	-1.58	0.141
	ATT	L	1.19	0.12	1.21	0.13	0.02	0.79	0.445
		R	1.20	0.14	1.18	0.10	-0.02	-0.65	0.532

**significant at*p*< 0.05; *trending at*p*< 0.1. Regions in bold indicate that the t-test was significant at*p*< 0.05 or trending at*p*< 0.10.

Hemi = hemisphere.

**Fig. 3.**
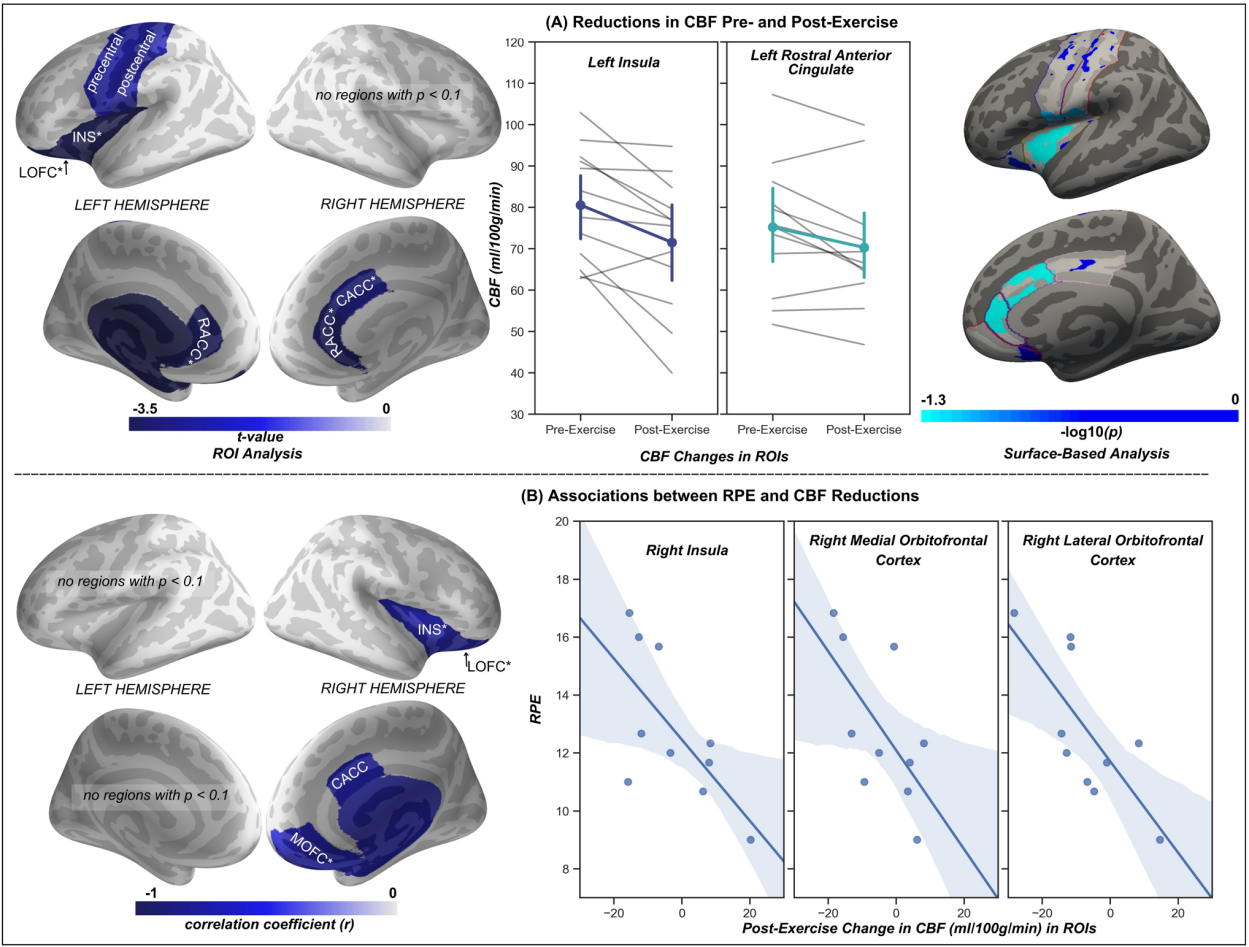
(A) Left panel: ROI analyses found that regions with decreased CBF (mL/100 g/min) post-exercise included left insular cortex (INS), lateral orbitofrontal cortex (LOFC), and rostral anterior cingulate cortex (RACC), as well as right rostral and caudal anterior cingulate (CACC). Regions with*p*< 0.1 shown, as well as regions with*p*< 0.05 (indicated with *). Center panel: Line plots of CBF changes in the left insula and rostral anterior cingulate shown. Grey lines represent individual participants. Colored lines represent average change across all participants, with error bars representing the 95% confidence interval for each timepoint. Right panel: Surface-based analyses found that clusters with decreased CBF post-exercise captured the left insular cortex and right rostral and caudal anterior cingulate. No regions survived comparison for multiple corrections, although marginally significant effects were observed in the left insula and right rostral and caudal anterior cingulate. (B) Left panel: Associations between post-exercise decreases in CBF and greater ratings of perceived exertion (RPE) during exercise were observed in the right insula, lateral orbitofrontal cortex, and right caudal anterior cingulate. Right panel: Association between post-exercise change in the right insula, medial orbitofrontal cortex, and lateral orbitofrontal cortex and ratings of perceived exertion. All shown associations significant at*p*< 0.05. precentral = precentral gyrus; postcentral = postcentral gyrus; PCC = posterior cingulate; MOFC = medial orbitofrontal cortex.

Surface-based analyses revealed that no regions showed significant change following correction for multiple comparisons. Two clusters demonstrated marginally significant decreases in CBF post-exercise (Fig. 3A), including clusters encompassing large portions of the left insula (cluster-wise*p*= 0.067) and right rostral and caudal anterior cingulate (cluster-wise*p*= 0.080).

There were no differences in motion, as assessed by mean framewise displacement, in pre-exercise and post-exercise ASL scans,*t*(11) = -0.24,*p*= 0.81.

### 3.4 Associations between cardiorespiratory fitness, ratings of perceived exertion, and exercise-induced changes in CBF

Increased RPE was associated with greater decreases in CBF in the right insular cortex (*r=*-0.68,*p=*0.032), right medial orbitofrontal cortex (*r=*-0.64,*p*= 0.045), and right lateral orbitofrontal cortex (*r=*-0.75,*p*= 0.013). A trending association was observed in the right caudal anterior cingulate (*r*= -0.60,*p*= 0.066) (Fig. 3B).

Post-hoc Pearson’s correlations were also used to assess the association between peak heart rate during exercise and CBF. Greater peak heart rate during exercise was found to be associated with larger decreases in CBF in the right insular cortex (*r*= -0.64,*p*= 0.024), but not other ROIs (*p*s > 0.1), including those regions showing greater decreases in CBF with increased RPE (right medial orbitofrontal cortex:*r*= -0.47,*p*= 0.124; right lateral orbitofrontal cortex:*r*= -0.47,*p*= 0.119; right caudal anterior cingulate:*r*= -0.26,*p*= 0.422).

A trending association between greater VO_2_peak and greater decreases in CBF pre- to post-exercise was observed in the right postcentral gyrus (*r=*0.52,*p=*0.08).

### 3.5 Arterial transit time

Example ATT images are shown inFigure 2. Longer ATT was observed post-exercise in two of the central command network regions examined: the right precentral gyrus and right postcentral gyrus (Table 1). A trending association between greater VO_2_peak and greater decreases in ATT pre- to post-exercise were identified in the left caudal anterior cingulate (*r=*-0.50,*p*= 0.097) and right postcentral gyrus (*r=*-0.52,*p*= 0.068). However, no significant changes in ATT were found in any other examined region and no surface-based clusters within central command regions showed significant changes following correction for multiple comparisons. No significant differences in regional ATT pre-exercise to post-exercise were associated with VO_2_peak or RPE in the examined central command regions.

## 4 Discussion

In the current feasibility study, we assessed changes in cerebral hemodynamics following light-to-moderate intensity exercise within cortical regions of the central command network. We used a pseudo-continuous ASL-MRI sequence with multiple post-labeling delays to mitigate potential partial volume effects, enable higher spatial resolution, and simultaneously examine two measures of cerebral hemodynamics, CBF and ATT. To our knowledge, ATT data have not been previously reported. We additionally used an “in-scanner” exercise stimulus protocol to reduce the delay between the exercise stimulus and the start of ASL-MRI data collection to about 90 seconds. We observed decreased CBF following exercise in the left insular cortex, left lateral orbitofrontal cortex, left and right rostral anterior cingulate cortex, and right caudal anterior cingulate cortex. Further, we found that the greater the subjective rating of perceived exertion, the larger the reduction in post-exercise CBF in the right insula, right medial orbitofrontal cortex, and right lateral orbitofrontal cortex. Reductions in CBF were associated with maximum heart rate during exercise blocks in the right insula. Finally, longer ATT was observed in the right precentral and right postcentral gyri. Cardiorespiratory fitness (VO_2_peak) was not associated with CBF or ATT in any examined region.

These findings suggest that light-to-moderate intensity exercise-related reductions in CBF within regions of the central command network can be detected by neuroimaging performed within 10 minutes of the exercise stimulus, that is, during the exercise recovery period. Notably, the regions of the central command network that showed the largest reductions in CBF following exercise were those regions thought to be primarily responsible for cardiovascular control (insular cortex, anterior cingulate, and medial prefrontal cortex;Shoemaker & Gros, 2024;Williamson, 2015;Williamson et al., 2001,^2002^,^2006^). On the other hand, central command regions governing motor control, such as the precentral and postcentral gyri (Shoemaker & Gros, 2024;Williamson, 2015), showed changes with ATT, but not CBF. Surface-based analyses provided preliminary additional support to the notion that the effect was specific and relatively consistent among these cardiovascular control regions, although these relationships were limited to marginally significant associations after multiple comparisons correction. Additional work with data acquisition during exercise and at multiple post-exercise delays with larger study samples may lend insight into this different pattern of CBF alterations in cardiovascular control and motor control regions of the central command network.

Our preliminary results indicate that perceived exertion may be associated with the magnitude of changes in CBF during exercise recovery. Greater CBF decreases in the cardiovascular control regions of the central command network, particularly in the right hemisphere, were associated with greater RPE (*p*s < 0.05). An objective measure of physical exertion, maximum heart rate, was not associated with CBF decreases in most of these regions, with the exception of right insular cortex. Prior work has hypothesized that cardiovascular control regions of the central command network are involved in not just feedforward processes, such as influencing heart rate and vessel dilation, but also feedback processes, including perceived exertion (Williamson, 2015;Williamson et al., 2006). BOLD activity in cardiovascular control regions of the central command network has been observed to increase with both performed exercise and imagined exercise (Thornton et al., 2001;Williamson et al., 2001,^2002^) and to be separable from muscular sensory input (Shoemaker, 2022). As such, it has been suggested that these regions use non-physical input, such as perceived exertion, to modulate cardiovascular output (Williamson, 2015;Williamson et al., 2006). Our finding that cardiovascular control regions of the central command network show decreased CBF in association with greater RPE but, in most regions, not maximum heart rate is consistent with this literature. This suggests that perceived exertion, in some cases above and beyond exertion itself, may be a critical component of cortical control of the cardiorespiratory system during exercise.

It is important to note, however, that participants in the present study exercised at a light-to-moderate intensity (average RPE = 12.8, SD = 2.6, corresponding to “somewhat hard” effort). There was variability among the ratings of exercise intensity among the participants (RPE range = 9 – 16.8, corresponding to “very light” to “very hard” efforts). No participant reported extremely hard or maximal exertion. Maximally vigorous exertion may have a different relationship with CBF during exercise recovery than observed in the current study. Prior work, for example, finds that CBF decreases back to baseline levels during vigorous exercise intensities (Braz & Fisher, 2016;González-Alonso et al., 2004;Hellstrom et al., 1996;Mulser & Moreau, 2023;Rooks et al., 2010), which may also influence CBF during recovery. Future work could further interrogate these relationships by including a larger sample size and prescribing multiple exercise intensities to further examine potential dose-response relationships between objective and subjective metrics of exertion and CBF.

Although it does not explain the specificity of findings within cortical control regions of the central command network, prior work has suggested that decreases in CBF during exercise recovery may be related to exercise-induced production of lactate and CO_2_, which enhances the release of oxygen from hemoglobin and therefore requires less CBF to maintain sufficient oxygen delivery (“Bohr-effect”;Bohr et al., 1904;Malte et al., 2021;Mast et al., 2022). Thus, exercising at higher intensities may enhance the release of oxygen from hemoglobin, thereby decreasing required CBF, as observed in the present study. Further, the observation of decreases in CBF during recovery is consistent with findings from prior studies (MacIntosh et al., 2014;Smith et al., 2010), includingMast et al. (2022), who reported reductions in global CBF and the hippocampus following moderate-to-vigorous exercise. Despite an equivalent sample size to the current study,Mast et al. (2022)reported greater decreases in CBF. For example, in the motor cortexMast et al. (2022)reported an average 17.5% decrease in CBF following exercise. In the present study, we observed an average 7.2% decrease in CBF in the left postcentral gyrus. These differences are most likely attributable to differences in exercise intensity between the two studies:Mast et al. (2022)directed participants to exercise at moderate and vigorous intensities, while participants within the present study exercised at a light-to-moderate intensity. Differences in effect size could be related to differences in exercise intensity and the strength of the Bohr-effect. Methodological differences in image acquisition may also have contributed to differences in effect size; for instance,Mast et al. (2022)voxel resolution was 37.8 mm^3^whereas our resolution was 15.6 mm^3^. Smaller voxel size can reduce partial volume effects and may impact CBF estimates. Nevertheless, although both studies had smaller sample sizes, the consistency in findings (decreased CBF) does provide some degree of confidence in the effect. Of course, additional studies with larger samples and, ideally, multiple exercise intensities sampled at multiple delays will be required to further elucidate the relationships between bouts of aerobic exercise and CBF.

A limited number of studies have examined ATT and, to our knowledge, the present study is the first to examine ATT following a bout of aerobic exercise. ATT and CBF are thought to capture complementary but unique characteristics of cerebral hemodynamics (Juttukonda et al., 2021;Liu et al., 2012). ATT is sensitive to a range of mechanisms that include not only cardiac output and slower blood velocity, but also path length (i.e., tortuosity) (Juttukonda et al., 2021). Thus, ATT can offer unique insight into cerebral hemodynamics after exercise. We found that ATTs increased following exercise in the right precentral and right postcentral gyrus, indicating slower transit times in these regions. As the present study lacks direct measurement of blood velocity, the mechanism behind the increased ATT is not clear. However, the ATT increases were observed in motor control regions, whereas the CBF reductions were observed in cardiovascular control regions, indicating that these modalities may characterize different exercise-induced alterations in cerebral hemodynamics.

One important consideration for the current protocol is scan timing. ASL acquisition is relatively slow (~5.5 minutes) and hemodynamics may change during that timeframe. This is an inherent limitation in the use of ASL to examine cerebral blood flow during recovery from exercise. A more controllable aspect of timing, however, is standardizing scan time between participants, such that imaging captures roughly equivalent pieces of each participant’s recovery process. Within the current design, ASL acquisition on average began 2 minutes, 32 seconds following exercise cessation (SD = 30 seconds). These differences are relatively small but could have introduced inter-subject differences that could impact results.

To summarize, we establish the feasibility of a novel exercise protocol that can be implemented in the bore of an MRI scanner and reduce the time delay between exercise and image acquisition. Our findings indicate post-exercise reductions in CBF in regions of the central command network, particularly within regions associated with cardiovascular control. A pre- versus post-exercise analysis of global gray matter CBF was not significant, supporting our ROI analysis that exercise-induced alterations in CBF are regionally specific. Whether or not this regional specificity of exercise-induced CBF changes is associated with potential domain-specific cognitive effects of exercise (Chang et al., 2012;Melville et al., 2024) was not examined, but it may be a contributing mechanism to exercise effects on cognitive performance. Exercise-induced reductions in CBF were associated with perceived exertion, including regions of the anterior cingulate cortex and insula, such that higher perceived exertion was associated with larger post-exercise reductions in CBF. Additional work is needed to further interrogate why there appears to be stronger associations between subjective perceptions of exertion and CBF compared to objective assessments of exertion, such as maximal heart rate. These results contribute to a burgeoning literature describing cerebral hemodynamics related to exercise. They suggest not only that reductions in cerebral blood flow occur immediately after exercise but also that these changes are commensurate with perceived exercise intensity. It is important to emphasize the timing of our data acquisition. The current results reflect post-exercise CBF changes and therefore reflect CBF changes during exercise recovery rather than CBF changes during exercise. Future studies should investigate these phenomena with larger sample sizes and across a range of exercise intensities, during exercise, and at multiple post-exercise delays to further elucidate both the dose-response relationship between exercise and CBF and any dynamic alterations in CBF during exercise and exercise recovery.

## Data Availability

Code is available underhttps://github.com/osubbal/ASL-CBF-Exercise_JCloud. For data access, please contact the corresponding author.
